# Incidence and risk of hypertension associated with vascular endothelial growth factor receptor tyrosine kinase inhibitors in cancer patients: a comprehensive network meta-analysis of 72 randomized controlled trials involving 30013 patients

**DOI:** 10.18632/oncotarget.11813

**Published:** 2016-09-01

**Authors:** Bo Liu, Fengxia Ding, Yang Liu, Geng Xiong, Tao Lin, Dawei He, Yuanyuan Zhang, Deying Zhang, Guanghui Wei

**Affiliations:** ^1^ Department of Urology, Children's Hospital of Chongqing Medical University, Chongqing, China; ^2^ Department of Respiratory Medicine, Children's Hospital of Chongqing Medical University, Chongqing, China; ^3^ Ministry of Education Key Laboratory of Child Development and Disorders, Chongqing Key Laboratory of Pediatrics, Chongqing International Science and Technology Cooperation Center for Child Development and Disorders, Chongqing, China; ^4^ Wake Forest Institute for Regenerative Medicine, Wake Forest School of Medicine, Winston-Salem, NC, USA

**Keywords:** meta-analysis, cancer, VEGFR-TKIs, hypertensive events

## Abstract

**Background:**

Tyrosine kinase inhibitors (TKIs) have been developed during the last decade that target the vascular endothelial growth factor receptor (VEGFR) are currently being evaluated as treatments for malignant tumors. The increased application of VEGFR-TKIs means that the probability of hypertension is a serious concern. However, the reported incidence varies markedly between clinical trials. Here, we undertook an up-to-date, comprehensive meta-analysis on clinical works to build the incidence of hypertension along with VEGFR-TKIs. The goal was to understand better of the overall venture of cancer patients’ hypertension treated with these drugs.

**Methods:**

Databases (EMBASE, PubMed, and Cochrane library) and the abstracts of the American Society of Clinical Oncology annual meeting and European Society of Medical Oncology were searched to identify related studies. 95% confidence intervals (CIs), summary incidences, and relative risk (RR) were calculated utilizing either fixed-effects models on the basis of the heterogeneity of the included studies or random-effects.

**Results:**

Seventy-two randomized controlled trials (including 30013 patients) were involved. The total incidence of high-grade and all-grade hypertensive events along with VEGFR-TKIs was 23.0% (95% CI, 20.1–26.0%) and 4.4% (95% CI, 3.7–5.0%), respectively. The use of VEGFR-TKIs remarkably enhanced the venture of developing high-grade (RR, 4.60; 95% CI, 3.92–5.40; *P* < 0.001) and all-grade (RR, 3.85; 95% CI, 3.37–4.40; *P* < 0.001) hypertensive events. Subgroup analyses revealed that the risk of a hypertensive event varied significantly in accordance with tumor type, VEGFR-TKI, trial phase, VEGFR-TKIs-based regimen, control therapy, and chemotherapy regimen.

**Conclusions:**

Patients with cancer that receive VEGFR-TKIs are at a remarkable venture of developing hypertension. Therefore, suitable treatment and monitoring should be introduced to avoid cardiovascular complications.

## INTRODUCTION

Malignant tumors are one of the most serious diseases threatening human life and for which systematic chemotherapeutics are still the main treatment of choice [1]. Because such treatments often fail due to the development of multidrug-resistant tumor cells, targeted therapies may be the best route forward [2]. Research and clinical practice show that vascular epithelial growth factor (VEGF) is very important for tumor growth, progression, and metastasis because it induces angiogenesis [3, 4]. Blockade of the VEGF signaling pathway is an important goal for those developing anti-cancer drugs [5]. Anti-VEGF monoclonal antibodies (e.g., bevacizumab) [6], anti-VEGF receptor (R)-2 antibodies (e.g., ramucirumab) [7], VEGF-ligand-binding fusion proteins (e.g., aflibercept) [8], and VEGFR-TKIs known as vascular epithelial growth factor receptor tyrosine kinase inhibitors (e.g., cabozantinib), the numerous angiogenesis inhibitors, have shown promising clinical efficacy against various malignant diseases and have been approved by the European Medicines Agency and the United States FDA [9].

Although VEGFR-TKIs are considered more specific and less toxic than conventional chemotherapy, severe side effects such as congestive heart failure and cerebrovascular events are particular concerns [10]. One major side effect noted in numerous trials is the onset of hypertension, the incidence of which ranges from 16.0-42.6% [11]. Adequate and aggressive treatment of hypertension is a significant issue for patients treated with VEGFR-TKIs due to the fact that serious kidney and cardiovascular diseases are caused by poorly controlled hypertension. Also, the usage of VEGF-TKIs could be linked to posterior reversible encephalopathy syndrome, which is a clinico-radiological event that includes symptoms such as headaches, nausea and emesis, visual loss and seizures, and particularly acute hypertension [12]. Not enough post-marketing experience, under-reporting, diagnosis difficulties, and bad follow-upping of exposed patients signify that the total risk and incidence of hypertension related to VEGFR-TKIs are uncertain. Thus, we performed this meta-analysis to examine the issue.

## RESULTS

### Systematic literature search

**Figure 1:**
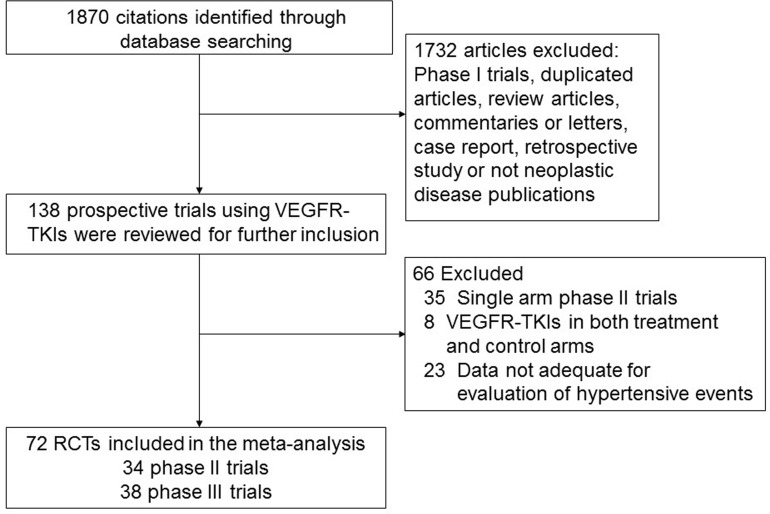
Selection process for randomized controlled trials included in the meta-analysis

The study was carried out according to the Systematic Review and Meta-analyses statement (Supplementary Table S2) [16]. For the meta-analysis, 1870 abstracts reported the usage of VEGFR-TKIs and 72 RCTs were comprised in total. Overall, 30013 patients were randomly assigned to either VEGFR-TKI-treated or control groups according to each individual trial eligibility criteria. The main criteria comprised of an Eastern Cooperative Oncology Group performance indicator of 0 or 1, adequate hematologic, cardiac, and kidney function, and a variety of cancers. Figure 1 describes the overall descriptive selection criteria. The attributes of the 72 incorporated trials was great [mean score, 4 (range, 3-5)]. Jadad scores of five were found thirty-five trials. Another 37 experiments did not describe the method for blinding and (or) randomization legibly; thus these trials were given scores of 3 or 4 on the Jadad scale. The patient and study attributes for all comprised trials are shown in Supplementary Table S3.

### Incidence of all-grade hypertensive events

The analysis contained 12736 patients treated with VEGFR-TKIs in 64 RCTs, 2972 of whom experienced hypertensive events. A renal cell Phase II trial carcinoma showed the highest overall incidence (64.2%; 95% CI, 51.2-77.1%) [54]; the three trials with the lowest incidence did not report any all-grade hypertensive events [33, 75, 85]. A random-effects model (c^2^-based Q-statistic test: q = 3727.72; *P* < 0.001; *I*^2^ = 98.3%) revealed that the total occurrence of all-grade hypertensive occasions in cancer patients treated with VEGFR-TKIs was 23.0% (95% CI, 20.1-26.0%, Supplementary Table 4 and Figure S1).

### Incidence of high-grade hypertensive events

The analysis contained 15975 patients treated with VEGFR-TKIs in 71 RCTs; 1023 patients experienced high-grade hypertensive events. The highest occurrence (30.8%; 95% CI, 26.7-35.0%) was seen in a Phase III trial for ovarian cancer [69], whereas the lowest overall incidence was detected in ten trials that reported no sign of high-grade hypertensive events [27, 33, 36, 46, 56, 62, 63, 75, 78, 85]. A random-effects model (c^2^-based Q-statistic test: q = 1065.86; *P* < 0.001; *I*^2^ = 93.4%) revealed that the total occurrence of all-grade hypertensive occasions in cancer patients treated with VEGFR-TKIs was 4.4% (3.7-5.0%, 95% CI, Supplementary Table 4 and Figure S2).

### RR of hypertensive events

23511 patients in 64 RCTs were incorporated when computing the RR of all-grade hypertensive events. The venture of all-grade hypertension occasions increased dramatically after treatment with VEGFR-TKIs: a random-effects model (*I*^2^ = 44.1, *P* < 0.001) yielded an RR of 3.85 (95% CI, 3.37-4.40; *P* < 0.001; Supplementary Table 4 and Figure S3). We also examined the stability and reliability of the combined results using a sensitivity analysis. The results showed that leaving any single trial out did not affect the significance estimate for the pooled RRs (Supplementary Figure S5 and Figure S6). Moreover, we conducted a meta-regression analysis to examine if different treatment times affected the RR of hypertensive events. Since 18 studies reported no data on the duration of the treatment, only 46 of the 64 studies were incorporated in the overall analysis. The results showed that different treatment times were not a source of heterogeneity (*P* = 0.896). High-grade hypertensive events occurred in a total of 29085 patients in 71 RCTs. The pooled RR derived from a fixed-effects model (*I*2 = 0%, *P* = 0.941) revealed that the danger of high-grade hypertensive incidents among patients of cancer was significantly higher after treatment with VEGFR-TKIs (RR, 4.60, 95% CI, 3.92-5.40; *P* < 0.001; Supplementary Table 4 and Figure S4).

### Risk of hypertensive events on basis of tumor type, VEGFR-TKI, trial phase, chemotherapy condition, treatment regimen, and control therapy

We next examined the RR of VEGFR-TKI-associated hypertensive events with regard to the classified tumor type. The largest RR of all-grade hypertensive occasions was found in individuals with breast cancer (95% CI, 2.96-12.79; RR, 6.15), while the smallest RR was detected in individuals with gastric cancer (95% CI, 0.02-43.40; RR, 0.88). Moreover, a markedly increasing danger of all-grade hypertensive occasions was detected in patients of HCC (RR, 3.04; 95% CI, 2.36-3.92), RCC (RR, 5.55; 95% CI, 2.75-11.19), thyroid cancer (RR, 4.61; 95% CI, 3.34-6.38), pancreatic cancer (RR, 3.22; 95% CI, 2.21-4.69), mCRC (RR, 4.05; 95% CI, 3.16-5.20), ovarian cancer (RR, 4.65; 95% CI, 2.30-9.42), GIST (RR, 2.93; 95% CI, 1.82-4.72), STS (RR, 5.38; 95% CI, 3.01-9.64), SCLC (RR, 2.38; 95% CI, 1.20-4.70), PENT (RR, 5.43; 95% CI, 1.96-15.08), and AML (RR, 2.21; 95% CI, 1.21-4.70). With respect to high-grade hypertensive events, the largest RR occurred in individuals with prostate cancer (RR, 8.85; 95% CI, 1.59-49.12), while the smallest was then detected in individuals with gastric cancer (RR, 0.88; 95% CI, 0.02-43.40). However, it was of interest to note that the danger of all-grade hypertensive events decreased non-significantly in patients with R/M HSNCC (RR, 0.94; 95% CI, 0.02-44.33) or gastric cancer (RR, 0.88; 95% CI, 0.02-43.40) treated with VEGFR-TKIs, and that the danger of high-grade hypertensive events decreased non-significantly in individuals with gastric cancer (RR, 0.88; 95% CI, 0.02-43.40). The RR of high-grade and all-grade cases are various significantly according to tumor type (*P* < 0.001), indicating that the probability of all-grade and high-grade hypertensive events after treatment of VEGFR varied in patients with different tumors.

The RR of hypertensive events caused by VEGFR-TKIs might be different. The largest RR of all-grade hypertensive events was detected in individuals treated with axitinib (RR, 9.17; 95% CI, 0.72-116.54), although it is not significantly different in this increased risk, while the smallest RR was detected in individuals treated with sorafenib (RR, 3.07; 95% CI, 2.43-3.87). The combined results also demonstrated that vandetanib (RR, 5.25; 95% CI, 4.12-6.70), sunitinib (RR, 7.91; 95% CI, 5.40-11.57), pazopanib (RR, 7.58; 95% CI, 3.08-18.62), cediranib (RR, 3.72; 95% CI, 2.95-4.70), regorafenib (RR, 3.96; 95% CI, 2.72-5.79), motesanib (RR, 4.02; 95% CI, 2.83-5.70), and cabozantinib (RR, 7.13; 95% CI, 2.97-17.15) led to a substantial increase in the risk of all-grade hypertensive events. With respect to high-grade hypertensive events, the highest RR was detected in patients receiving cabozantinib (RR, 9.17; 95% CI, 1.24-67.77), while the smallest was detected in individuals receiving motesanib (RR, 1.01; 95% CI, 0.02-50.87). A remarkably increasing risk was detected as well in those taking sorafenib (RR, 3.66; 95% CI, 2.89-4.63), vandetanib (RR, 5.85; 95% CI, 3.36-10.20), sunitinib (RR, 4.35; 95% CI, 3.12-6.07), pazopanib (RR, 5.06; 95% CI, 3.55-7.22), cediranib (RR, 6.13; 95% CI, 3.43-10.97), axitinib (RR, 4.22; 95% CI, 1.75-10.16), and regorafenib (RR, 7.81; 95% CI, 3.06-19.94). The RR of high-grade and all-grade hypertensive cases varied significantly according to the type of VEGFR-TKI (*P* < 0.001); thus the risk of high-grade and all-grade hypertensive events probably differs according to the VEGFR-TKI prescribed.

Next, we executed a subgroup examination of the trial phase. The data manifested that the RR of all-grade hypertensive events for Phase II trials was 3.43 (95% CI, 2.66-4.42) *versus* 4.06 (95% CI, 3.48-4.74) for Phase III trials, whereas the RR of high-grade hypertensive events for Phase II trials was 3.28 (95% CI, 2.31-4.66) *versus* 4.97 (95% CI, 4.14-5.96) for Phase III trials. This diversity between the risk of high-grade and all-grade hypertensive events at different test phases was statistically significant (*P* < 0.001). We also conducted a subgroup examination stratified based on a chemotherapy regimen. The results manifested the RR of all-grade hypertensive events for chemotherapy-naïve patients was 3.33 (95% CI, 2.82-3.94) whereas that for pre-chemotherapy was 4.36 (95% CI, 3.57-5.33). The RR of high-grade hypertensive events for chemotherapy-naïve patients was 3.87 (95% CI, 3.12-4.81) *versus* 5.61 (95% CI, 4.40-7.16) for pre-chemotherapy patients. This diersity between the risk of high-grade and all-grade hypertensive events according to chemotherapy conditions was statistically significant (*P* < 0.001). Next, we conducted a subgroup investigation based on the VEGFR-TKI-based regimen. The all-grade hypertensive occurrences in patients received VEGFR-TKI monotherapy RR was 4.49 (95% CI, 3.74-5.39) *versus* 3.24 (95% CI, 2.70-3.88) for those receiving combination therapy. Meanwhile, the RR of high-grade hypertensive occurrences for those receiving VEGFR-TKI monotherapy was 5.29 (95% CI, 4.30-6.51) *versus* 3.80 (95% CI, 2.96-4.88). The difference between the danger of high-grade and all-grade hypertensive occurrences according to VEGF-TKI regimen was statistically significant (*P* < 0.001). Finally, we performed subgroup analysis based on control therapy. The pooled analysis demonstrated that treatment with a VEGFR-TKI was accompanied by a much higher risk of all-grade hypertensive cases than the placebo (RR, 4.16; 95% CI, 3.47-4.98) or non-placebo (RR, 3.63; 95% CI, 2.98-4.41) therapy. In addition, our analysis revealed a large increase in the risk of high-grade hypertensive events when patients received VEGFR-TKIs rather than placebo (RR, 5.13; 95% CI, 4.13-6.38) or non-placebo (RR, 3.81; 95% CI, 3.01-4.83) therapy. These differences were statistically significant (*P* < 0.001).

**Figure 2:**
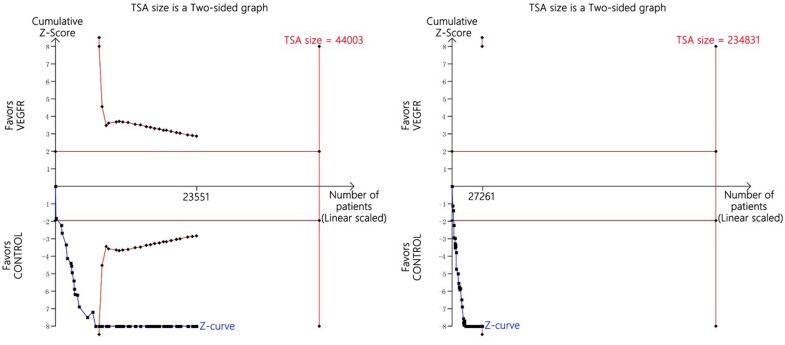
Trial sequential analysis of trials with a lower risk of bias when reporting hypertensive events

We used TSA to ensure that suitable sample sizes were included to make sure that the results were not influenced by newly published research, although the cumulative information size did not reach the satisfied information volum; however, the cumulative Z-curve crossed the cut-off level, so we concluded that no additional trials were needed (Figure 2).

### Publication bias

**Figure 3:**
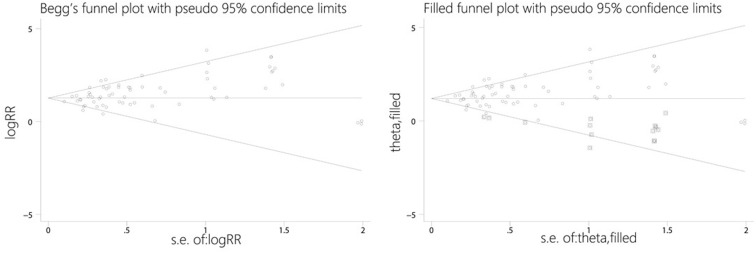
Funnel plots without and with trim and fill

Funnel plots and Egger's tests detected clear publication bias with respect to the RR of the all-grade hypertensive events (*P* < 0.001). So, we adopted an iterative method to evaluate the number of deficient studies (Trim and fill method) and then performed a new meta-analysis after adding in some hypothetical trials [88]. The results still showed a statistically significant relationship between the use of VEGFR-TKIs and all-grade hypertension (95% CI, 3.00-3.97; RR, 3.45), indicating that the results are unlikely to be affected by publication bias (Figure 3). Egger's tests revealed no significant publication bias with respect to the RR of high-grade hypertensive events (*P* = 0.312). With the trim and fill method, the result also showed a significant association between use of VEGFR-TKIs and hypertension (RR, 4.13; 95% CI, 3.51-4.87).

## DISCUSSION

VEGF is very important for tumor growth, progression, and metastasis because it induces angiogenesis [3, 4]. During the last century, Folkman proposed that new blood vessel formation or neovascularization may be a new paradigm for tumorigenesis [89, 90]. Since then, several anti-angiogenesis drugs that inhibit angiogenesis and malignancy have been ratified for usage as agents to aid with cancer therapy[25, 26, 53, 58, 74]. However, extensive clinical trials of angiogenesis inhibitors used to treat cancer revealed that these inhibitors have unexpected side effects and that hypertensive events are one of the most common. Another meta-analysis indicated that the incidence of hypertension among patients receiving bevacizumab (an anti-VEGF antibody) was 8% (95% CI, 6-10%), and that the danger for the onset of hypertension in individuals treated with bevacizumab increased significantly (RR, 5.38; 95% CI, 3.63-7.97) [91]. Nevertheless, it is unclear if the usage of VEGFR-TKIs that target VEGF signaling pathways increases the chance for the onset of hypertension in cancer patients.

We believe that this review is one of the first and largest meta-analysis to assess the relationship between hypertension and VEGFR-TKIs. We attempted to examine the risk of hypertension during treatment with various TKIs using CTCAE 2.0 or 3.0. The collective occurrence of VEGFR-TKI-associated high-grade and all-grade hypertensive cases was 23.0% (95% CI, 20.1-26.0%) and 4.4% (95% CI, 3.7-5.0%), respectively, which is lower than that formerly published by Wu (all-grade, 23.4%; high-grade, 5.7%) [92]. One possible explanation for this discrepancy is a difference in the tumor type distribution: the major type of cancer examined by Wu was RCC (about 44.4% of cases), whereas RCC accounted for only 6.3% of the cases in the present study. Also, Wu examined a small number of RCTs (only four trials). Therefore, the collective study might have been altered by the results of a single large RCT. We also found that VEGFR-TKIs caused a significant increase in the probability of all-grade hypertensive events (RR, 3.85; 95% CI, 3.37-4.40; *P* < 0.001) and high-grade hypertensive events (RR, 4.60; 95% CI, 3.92-5.40; *P* < 0.001). It was indicated by sensitivity analysis that leaving out any single trial had an insignificant effect on the collective all-grade and high-grade RRs. Based on these results, we concluded that VEGFR-TKIs dramatically enhance the occurrence of hypertension in patients of cancer and that intensive monitoring for VEGFR-TKI-associated hypertensive events is suggested throughout the management of hypertension. Furthermore, VEGFR-TKIs appear to increase the risk of renal and cardiovascular events. Appropriate monitoring and management of blood pressure (BP) are expected to reduce both mortality and morbidity due to renal insufficiency, congestive heart failure, stroke, and myocardial infarction, and may prevent patients from abandoning treatment [93].

Additionally, we explored the factors that might lead to risk for VEGFR-TKI-associated hypertensive events. We found that the risk of hypertensive events related to VEGFR-TKIs varies mainly based on tumor type. This finding could be due to the fact that different malignancies have different pathogeneses and different spectra of patient comorbidities. Patients with different tumor types that were treated with VEGFR-TKIs were at a high risk of hypertensive events; the two exceptions were R/M HNSCC and gastric cancer. A possible explanation for this is that the control therapies used in these two cases, cetuximab and docetaxel, are also related with an increased chance of hypertension [94, 95]. This may reduce the RR of VEGFR-TKI-associated hypertension. The occurrence of hypertension connected to different VEGFR-TKIs was also examined, and the results showed that sunitinib (RR, 7.91; 95% CI, 5.40-11.57), pazopanib (RR, 7.58; 95% CI, 3.08-18.62), cabozantinib (RR, 7.13; 95% CI, 2.97-17.15), vandetanib (RR, 5.25; 95% CI, 4.12-6.70), motesanib (RR, 4.02; 95% CI, 2.83-5.70), regorafenib (RR, 3.96; 95% CI, 2.72-5.79), cediranib (RR, 3.72; 95% CI, 2.95-4.70), and sorafenib (RR, 3.07; 95% CI, 2.43-3.87) led to a remarkable increment in the risk for all-grade hypertensive events. Cabozantinib (RR, 9.17; 95% CI, 1.24-67.77), regorafenib (RR, 7.81; 95% CI, 3.06-19.94), cediranib (RR, 6.13; 95% CI, 3.43-10.97), vandetanib (RR, 5.85; 95% CI, 3.36-10.20), pazopanib (RR, 5.06; 95% CI, 3.55-7.22), sunitinib (RR, 4.35; 95% CI, 3.12-6.07), axitinib (RR, 4.22; 95% CI, 1.75-10.16), and sorafenib (RR, 3.66; 95% CI, 2.89-4.63) also increased the probability of high-grade hypertensive events. The probability of hypertensive cases associated with VEGFR-TKIs varied significantly among individuals receiving varying types of VEGFR-TKI; this may be because different VEGFR-TKIs target different receptors. Another potential hazard is probably simultaneous treatment with VEGFR-TKIs and other drugs. Our analysis demonstrated that an increased RR of high-grade and all-grade hypertensive events was significantly associated with both VEGFR-TKI monotherapy and combination therapy.

The VEGF pathway has an essential role in multiple physiological processes, including vascular and cardiomyocyte homeostasis, tissue neovascularization, and wound healing [96–98]. The mechanisms by which VEGFR-TKIs elevates BP remain uncertain. Evidence implies that the effects may be related directly to inhibition of the VEGF receptor. Such effects may include 1) impairment of angiogenesis, which reduces microvessel density (rarefaction); 2) production of molecules in response to hypoxia, which leads to an increase in vascular tone; 3) endothelial cell dysfunction, which leads to increased peripheral resistance; and 4) alterations in neurohormonal factors or the rennin-angiotensin-aldosterone system [99, 100]. Nevertheless, few studies have explored the potential mechanisms underlying hypertension related to VEGFR-TKIs. Therefore, future studies centered around these specific issues are needed.

Although VEGFR-TKI-induced hypertension is a common adverse effect noticed by oncologists and cardiovascular medicine specialists, the deterrence and supervision of cardiovascular toxic effects remain controversial. Based on the guidelines of the NCI [101], the goal BP for individuals accepting anti-VEGF therapy is less than 140/90 mm Hg. For some patients at high risk of cardiovascular complications, targets should be revised downward. Before anti-VEGF therapy, BP should be well-controlled for more than 1 week. NCI clinical trial protocols advise that BP be monitored on a weekly basis in the time of the first cycle of anti-VEGF therapy, following by at least every two to three weeks throughout treatment. If stage 1 hypertension (≥140/90 mm Hg) occurs or diastolic pressure increases by 20 mm Hg from baseline during treatment, then anti-hypertensive therapy is required, the dose of current anti-hypertensive drugs should be adjusted, or new anti-hypertensive drugs should be added to ensure better control. Many drugs can be used to treat hypertension caused by VEGFR-TKIs. Non-dihydropyridine calcium-channel antagonists like diltiazem and verapamil are cytochrome P4503A4 (CYP3A4) inhibitors. Other drugs include dihydropyridines like nifedipine and amlodipine. Alternatively, ACEI or ARB are reasonable choices; these drugs have the added merits of enhancing endothelial function and microvessel density. Besides, antihypertensive medications like alpha blockers, diuretics and beta blockers, can also be utilized to control hypertension caused by VEGFR-TKIs [101, 102]. However, it is unclear whether any one agent is superior to another. When determining the type of antihypertensive medication to be utilized for a patient, the individual's medical condition and health status should be taken into consideration.

Despite our efforts to minimize the effects of confounding variables, there are several limitations that need to be considered. First, the incorporated trials were performed by different researchers from various institutions; thus the reported incidence of hypertensive events may suffer from potential bias. Also, the varying types of tumors and various VEGFR-TKIs examined might enhance heterogeneity. Moreover, the side effects often times will depend on the specific type of tumor in question. One such example includes RCC patients with nephrectomy and/or renal dysfunction who were more vulnerable to hypertension after treatment of VEGFR-TKIs. Second, we examined a diverse population of individuals receiving VEGFR-TKI monotherapy or VEGFR-TKI-based combination therapy. Therefore, the design of the treatment in all arms was not exactly the same; moreover, the completeness of follow-up might lead to the root of heterogeneity. Third, pre-existing hypertension that have been controlled was more likely to influence the occurrence of hypertensive events during treatment, although patients with uncontrolled hypertension are generally excluded from VEGFR-TKI trials. Thus, the incidence and risk of hypertension might have been overestimated in our analysis. Therefore, in order to clarify this issue more trials are needed. Finally, our analysis was based on clinical trial levels rather than on individual patient data; therefore, confounding variables (e.g., comorbidities) were not included. Nevertheless, the trials incorporated in our analysis were high quality; some studies suggest that there are no significant differences between meta-analyses carried out at the trial level or individual level [103].

In summary, our RCTs investigation revealed the usage of VEGFR-TKIs is related to an enhanced occurrence of high-grade and all-grade hypertensive cases. Also, the probability of hypertension varies in line with tumor type and the type of VEGFR-TKI used. This information will assist physicians with recognizing the probability of hypertension related to VEGFR-TKIs and will help to tailor both dose and schedule to suit individual patients.

## MATERIALS AND METHODS

### Data sources

Publications from PubMed (January 1, 1966, to February 29, 2016) were reviewed. The search method is composed of the following items: sorafenib, BAY 43-9006, nexavar, AZD2171, sutent, SU11248, votrient, sunitinib, GW786034, vandetanib, ZD6474, caprelsa, dovitinib, nintedanib, ZD6474, axitinib, cediranib, regorafenib, BAY 73-4506, linifanib, ABT-869, motesanib, AMG 706, pazopanib, AG-013736, cabozantinib, cancer, and hypertension. Randomized, prospective, and controlled clinical trials composed the analysis. Key words such as hypertension, angiogenesis inhibitors, and VEGF were used to identify related papers. Additionally, objective searches of EMBASE (data from June 26, 1980 to February 29, 2016) and the Cochrane library were performed to guarnatee that no applicable clinical trial were ignored. Meanwhile, the website http://www.ClinicalTrials.gov was investigated in order to obtain more relevant registered research. Finally, trials reported at ASCO (http://asco.org/ASCO) and ESMO (http://www.esmo.org/ESMO) from 2001 to 2015 were also examined. If duplicate trials were identified, the latest trials with more detailed data were included.

### Quality assessment and data extraction

The quality of each eligible study was assed using the five-point Jadad ranking system: a score of three or above in a trial was esteemed as high quality [13]. Data extraction was performed impartially by two researchers (BL and FD), and any differences were decided based on discussion. The required information was isolated from all of the studies. To ensure clinical significance, the trials in Phase I were not included in the study due to dose disparities in the data and small sample sizes. The selection criteria were as follows: (1) Phase II and Phase III RCTs involving cancer patients; (2) cancer patients had received VEGFR-TKIs or control treatments (placebo, current chemotherapy, best supportive care, and care standard); and (3) safety data with respect to hypertension events and sample sizes were available.

### Clinical endpoints

The safety profile of each trial were analyzed, where clinical endpoints were selected. Events caused by hypertension were recorded in line with National Cancer Institute (NCI) CTCAE 2.0 or 3.0 (ctep.cancer.gov); both versions are identical with respect to the grading of hypertension (which starts at grade 1) (Supplementary Table S1).

### Statistical analysis

We utilized Stata version 12.0 (Stata Corporation, College Station, TX) to analyze the data. To calculate the overall incidence of hypertensive events, the quantity of patients with high-grade and all-grade hypertensive events and the quantity of patients exposed to VEGFR-TKIs were abstracted. The the 95% confidence interval (CI) for all-grade and high-grade hypertensive patients and relative risk (RR) were then computed. Half-integer continuity correction was used if zero events were reported in the arm [14]. Statistical heterogeneity between trials was evaluated utilizing the χ^2^ test [15]. Heterogeneity was esteemed statistically significant when P_heterogeneity_ < 0.1. A fixed-effects or random-effects model was utilized according to whether heterogeneity existed or not. *P* values <0.05 (two-tailed) were considered significant. Subgroup evaluation was performed when heterogeneity existed. Sensitivity was analysed to test the stability of the obtained data, and we utilized Egger's test to evaluate publication bias.

### Trial sequential analyses

Trial sequential analyses (TSAs) were conducted to measure the possibility of type I error and to help evaluate the need for expanding the sample size. If zero events were reported in the trials, an additional 0.5 event was included in the arms. Two-sided tests were used, and a type I error was put at 5% and power at about 80%. The incidence of all-grade and high-grade hypertensive events in the control experiment was put at 3.7% and 0.3%, respectively. TSA was conducted using TSA V.0.9 β (please visit www.ctu.dk/tsa/).

## SUPPLEMENTARY MATERIAL FIGURES AND TABLES










