# Supplementary material for: Incidence and risk of hypertension associated with vascular endothelial growth factor receptor tyrosine kinase inhibitors in cancer patients: a comprehensive network meta-analysis of 72 randomized controlled trials involving 30013 patients
Source: Oncotarget. 2016 Sep 1;7(41):67661–73. doi: 10.18632/oncotarget.11813 (PMC5341903; doi:10.18632/oncotarget.11813)
Supplement: Supplementary file 1 [file oncotarget-07-67661-s001.pdf]

Incidence and risk of hypertension associated with vascular endothelial growth factor receptor tyrosine kinase inhibitors in cancer patients: a comprehensive network meta-analysis of 72 randomized controlled trials involving 30013 patients

Supplementary Material

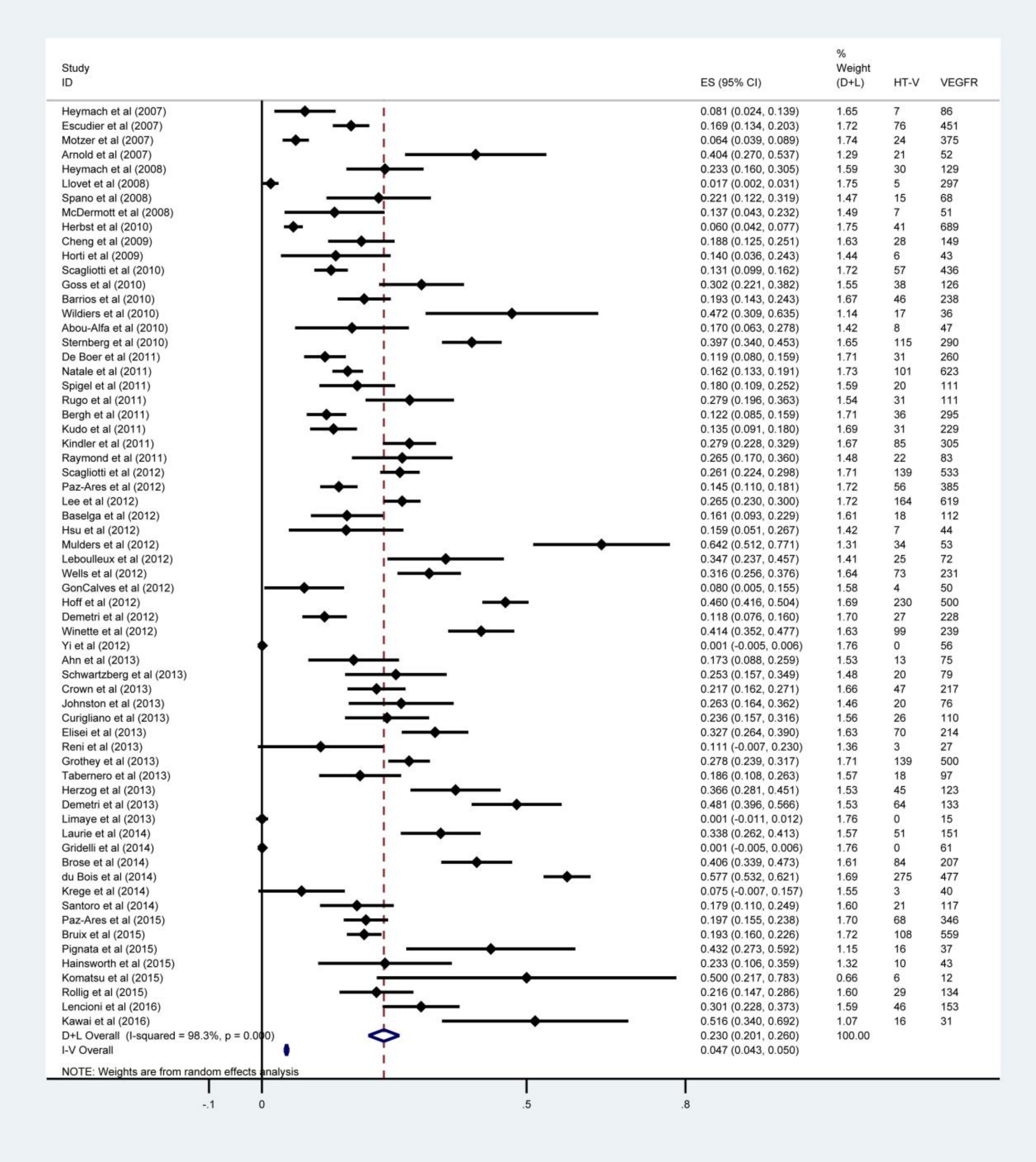

Figure S1. Incidence of all-grade hypertension with VEGFR-TKIs.

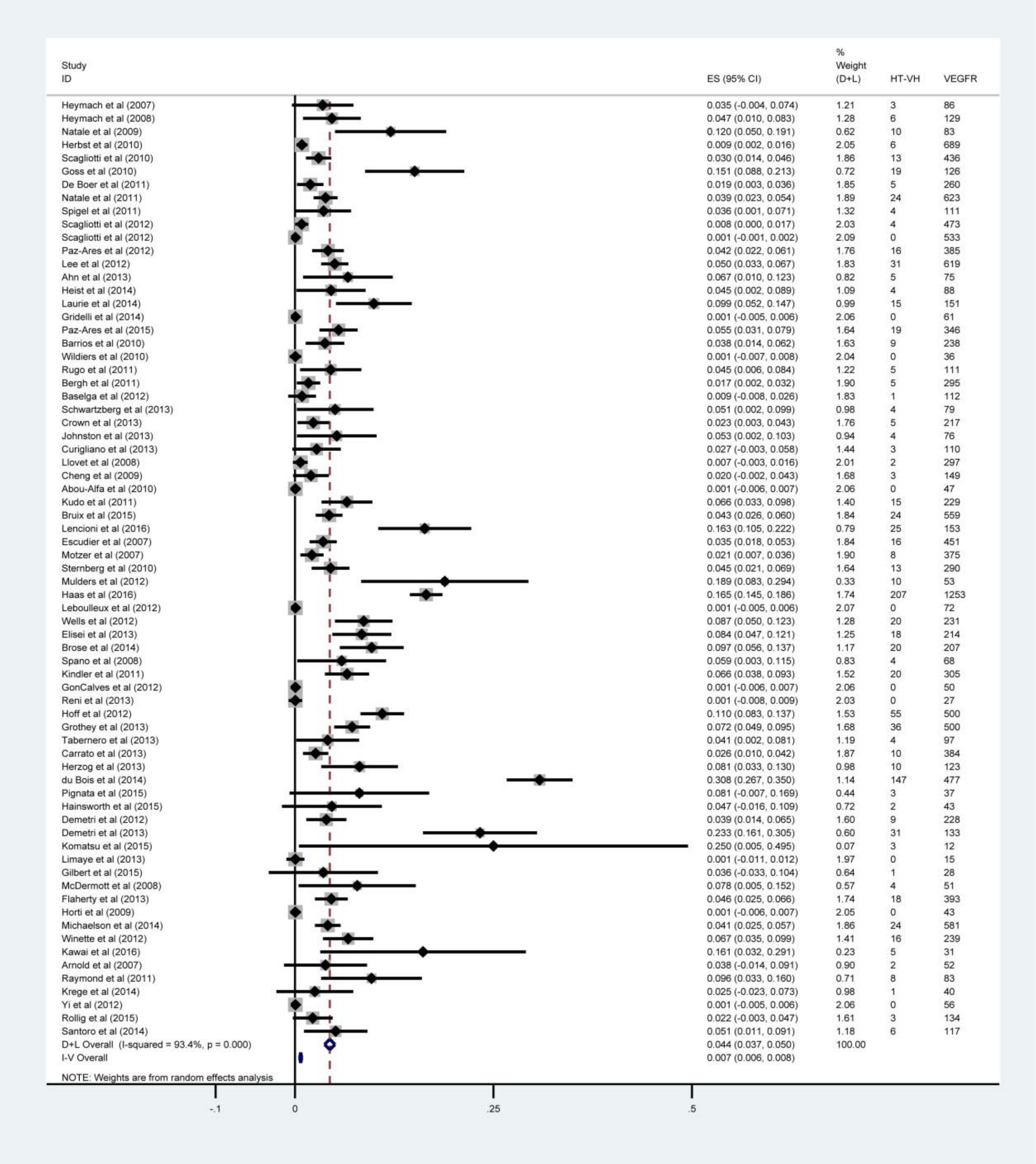

Figure S2. Incidence of high-grade hypertension with VEGFR-TKIs.

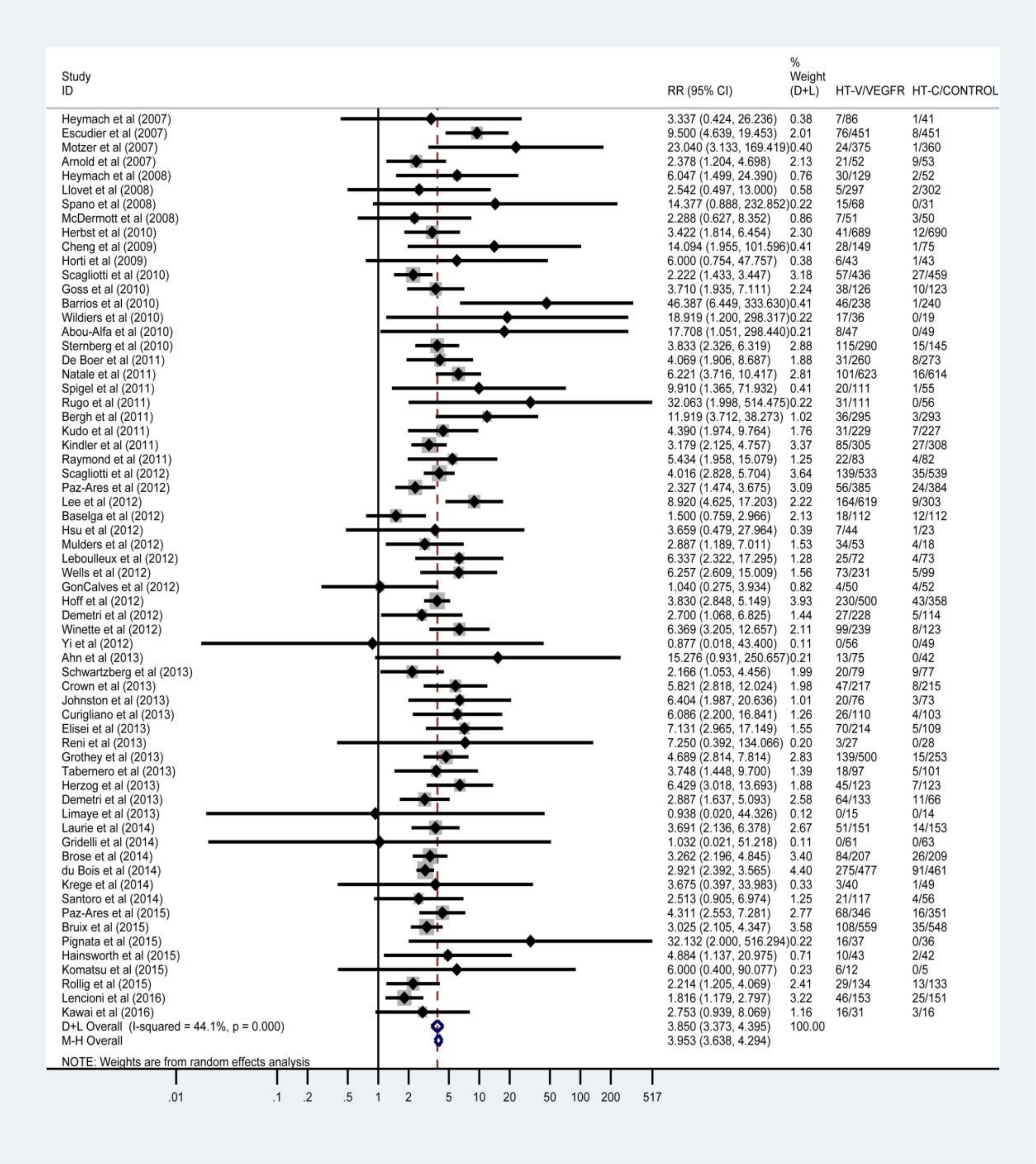

Figure S3. The relative risk of all-grade hypertension with VEGFR-TKIs versus control.

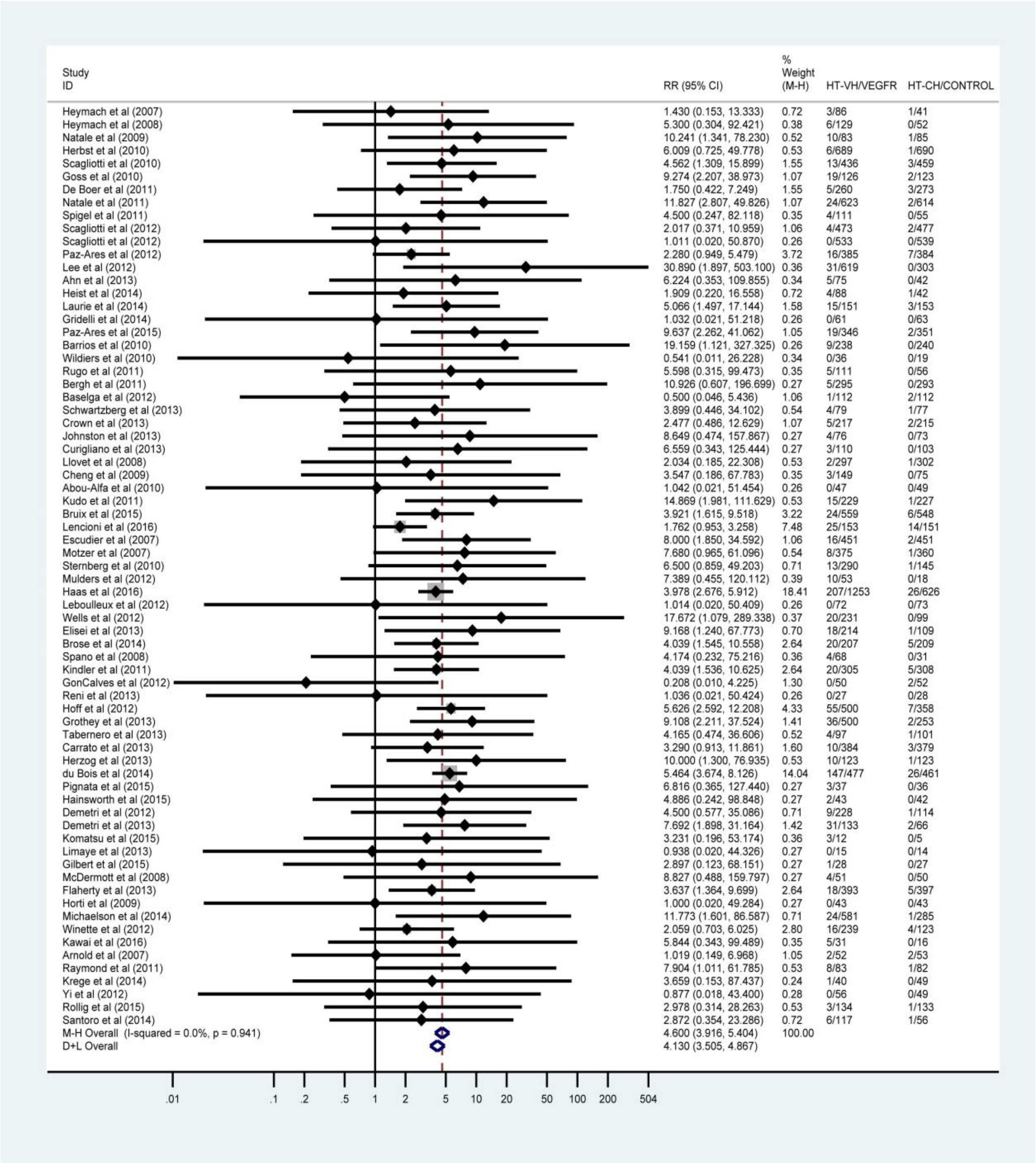

Figure S4. The relative risk of high-grade hypertension with VEGFR-TKIs versus control.

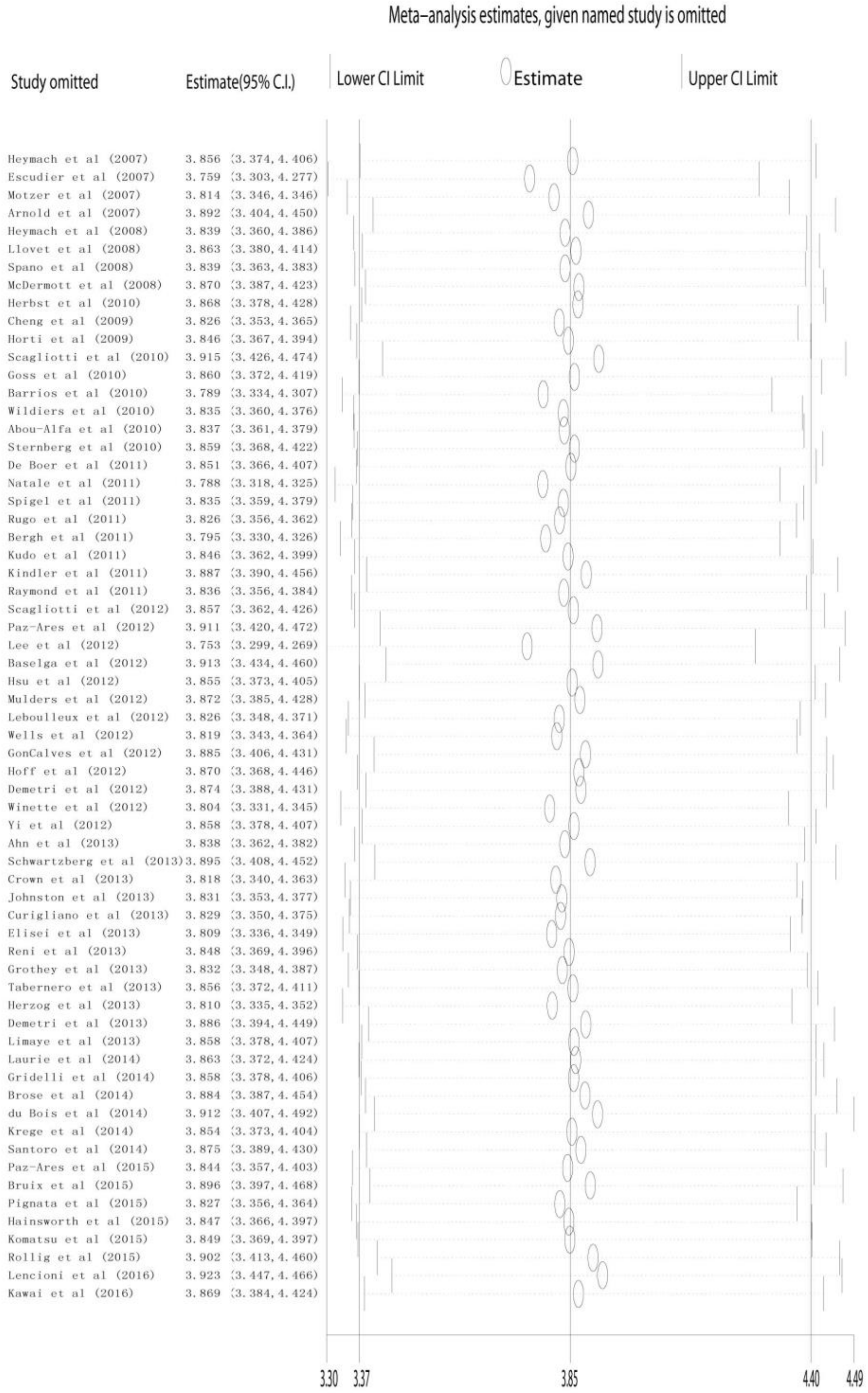

Figure S5. Meta-analysis of all-grade hypertensive events associated with VEGFR-TKIs versus control: ‘leave-one-out’ sensitivity analysis

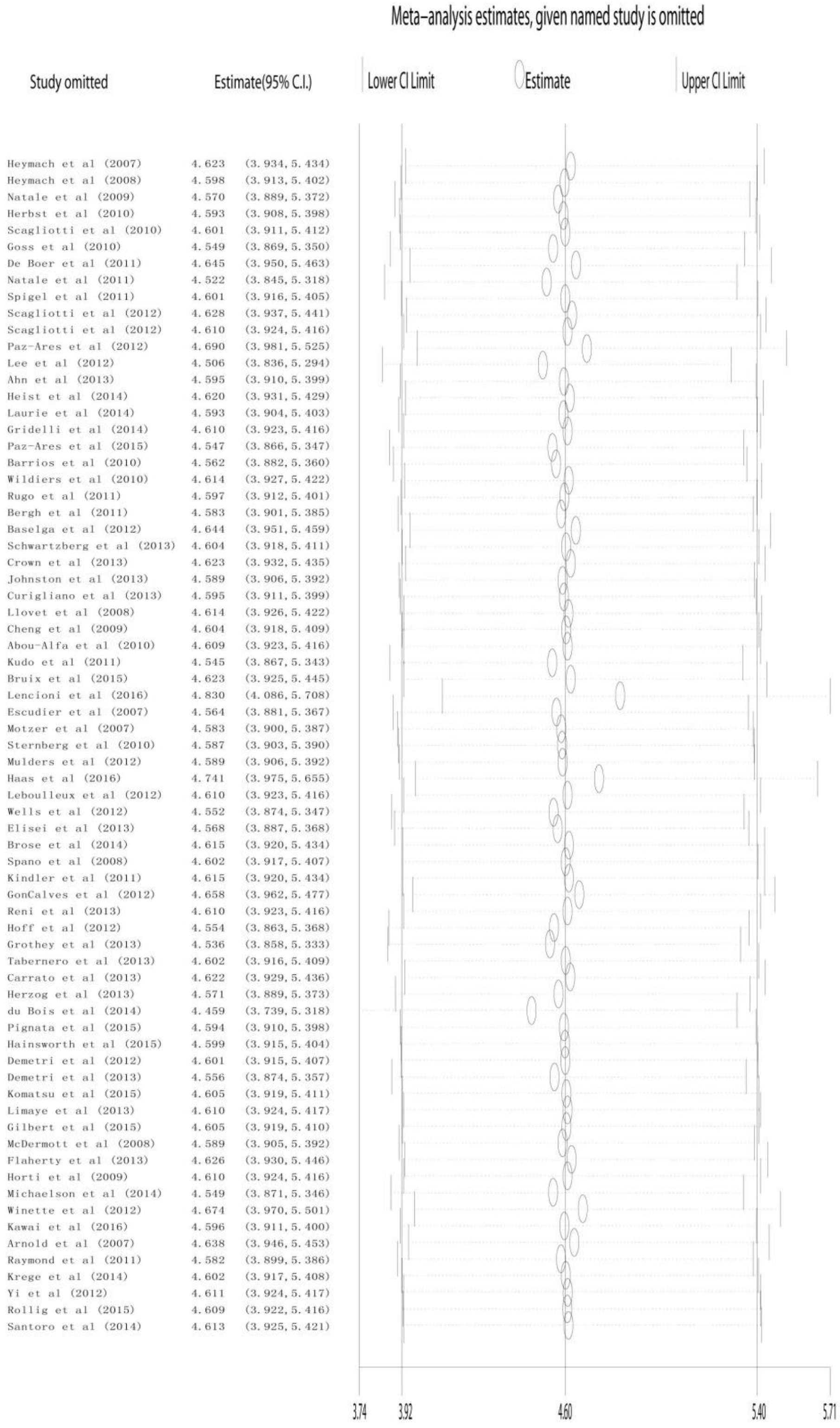

Figure S6. Meta-analysis of high-grade hypertensive events associated with VEGFR-TKIs versus control: 'leave-one-out' sensitivity analysis
