# Supplementary material for: Incidence and risk of hypertension associated with vascular endothelial growth factor receptor tyrosine kinase inhibitors in cancer patients: a comprehensive network meta-analysis of 72 randomized controlled trials involving 30013 patients
Source: Oncotarget. 2016 Sep 1;7(41):67661–73. doi: 10.18632/oncotarget.11813 (PMC5341903; doi:10.18632/oncotarget.11813)
Supplement: Supplementary file 2 [file oncotarget-07-67661-s002.doc]

**Supplemental Table 1.** National Cancer Institute CTCAE (versions 2 and 3) for hypertension

| **Grade** | **Version 2.0** | **Version 3.0** |
| --- | --- | --- |
| **1** | An asymptomatic, transient increase in DBP by >20 mm Hg or an increase in BP to >150/100 mm Hg if previously WNL. No treatment required. | An asymptomatic, transient (<24 h) increase in DBP by >20 mm Hg or an increase in BP to >150/100 mm Hg if previously WNL. No intervention indicated. |
| **2** | Recurrent, persistent, or symptomatic increase in DBP by >20 mm Hg or an increase in BP to >150/100 mm Hg if previously WNL. No treatment required. | Recurrent, persistent (≥24 h), or symptomatic increase in DBP by >20 mm Hg or an increase in BP to >150/100 mm Hg if previously WNL. Monotherapy may be indicated. |
| **3** | Requiring therapy or more intensive therapy than previously. | Requiring more than one drug or more intensive therapy than previously. |
| **4** | Hypertensive crisis | Life-threatening consequences (e.g., hypertensive crisis). |
| **5** | - | Death |

BP, blood pressure; DBP, diastolic blood pressure; WNL, within normal limits.
