# Supplementary material for: Incidence and risk of hypertension associated with vascular endothelial growth factor receptor tyrosine kinase inhibitors in cancer patients: a comprehensive network meta-analysis of 72 randomized controlled trials involving 30013 patients
Source: Oncotarget. 2016 Sep 1;7(41):67661–73. doi: 10.18632/oncotarget.11813 (PMC5341903; doi:10.18632/oncotarget.11813)
Supplement: Supplementary file 4 [file oncotarget-07-67661-s004.doc]

**Supplemental Table 3.** The 72 randomized controlled trials (RCTs) included in the meta-analysis

| Tumor types | Study | Phase | Random patients (*n*) | Therapeutic regimen | Median age (years) | Median treatment duratio*n* (months) | Median PFS/TTP (months) | Patients for analysis | Reported hypertensive events  All-grade (High-grade) | Jadad score |
| --- | --- | --- | --- | --- | --- | --- | --- | --- | --- | --- |
| NSCLC | Heymach et al. (2007) [17] | II | 127 | Vandetanib 300 mg + TXT | 61 | NR | 17 weeks | 44 | 4 (2) | 3 |
|  |  |  |  | Vandetanib 100 mg + TXT | 58 | NR | 18.7 weeks | 42 | 3 (1) |  |
|  |  |  |  | Placebo + TXT | 60 | NR | 12 weeks | 41 | 1 (1) |  |
|  | Heymatch et al. (2008) [18] | II | 181 | Vandetanib 300 mg + CP | 60 | NR | 24 weeks | 56 | 18 (4) | 3 |
|  |  |  |  | Vandetanib 300 mg | 63 | NR | 11.5 weeks | 73 | 12 (2) |  |
|  |  |  |  | Placebo + CP | 59 | NR | 23.1 weeks | 52 | 2 (0) |  |
|  | Natale et al. (2009) [19] | II | 168 | Vandetanib 300 mg | 63 | NR | 11 weeks | 83 | (10) | 5 |
|  |  |  |  | Gefitinib | 61 | NR | 8.1 weeks | 85 | (1) |  |
|  | Herbst et al. (2010) [20] | III | 1391 | Vandetanib 100 mg + TXT | 59 | 12.1 weeks | 4 | 689 | 41 (6) | 5 |
|  |  |  |  | Placebo + TXT | 59 | 13 weeks | 3.2 | 690 | 12 (1) |  |
|  | Scagliotti et al. (2010) [21] | III | 926 | Sorafenib 400 mg + CP | 62 | 16.6 weeks | 4.6 | 436 | 57 (13) | 3 |
|  |  |  |  | Placebo + CP | 63 | 17.9 weeks | 5.4 | 459 | 27 (3) |  |
|  | Goss et al. (2010) [22] | II | 296 | Cediranib 30 mg + CP | 60 | 20 weeks | 5.6 | 126 | 38 (19) | 5 |
|  |  |  |  | Placebo + CP | 58 | 19 weeks | 5 | 123 | 10 (2) |  |
|  | De Boer et al. (2011) [23] | III | 534 | Vandetanib 100 mg + pemetrexed | 60 | 102 days | 17.6 weeks | 260 | 31 (5) | 5 |
|  |  |  |  | Placebo + pemetrexed | 60 | 85 days | 11.9 weeks | 273 | 8 (3) |  |
|  | Natale et al. (2011) [24] | III | 1240 | Vandetanib 300 mg | 61 | 9.1 weeks | 2.6 | 623 | 101 (24) | 3 |
|  |  |  |  | Erlotinib | 61 | 8.6 weeks | 2.0 | 614 | 16 (2) |  |
|  | Spigel et al. (2011) [25] | II | 168 | Sorafenib 400 mg + erlotinib | 65 | NR | 3.38 | 111 | 20 (4) | 5 |
|  |  |  |  | Placebo + erlotinib | 65 | NR | 1.94 | 55 | 1 (0) |  |
|  | Scagliotti et al. (2012) [26] | III | 960 | Sunitinib 37.5 mg + erlotinib | 61 | 12 weeks | 3.6 | 473 | (4) | 5 |
|  |  |  |  | Placebo + erlotinib | 61 | 12 weeks | 2.0 | 477 | (2) |  |
|  | Scagliotti et al. (2012) [27] | III | 1090 | Motesanib 125 mg + CP | 60 | 4.1 | 5.6 | 533 | 139 (0) | 5 |
|  |  |  |  | Placebo + CP | 60 | 4.1 | 5.4 | 539 | 35 (0) |  |
|  | Paz-Ares et al. (2012) [28] | III | 904 | Sorafenib 400 mg + GC | 60 | 17 weeks | 6.0 | 385 | 56 (16) | 5 |
|  |  |  |  | Placebo + GC | 58 | 18 weeks | 5.5 | 384 | 24 (7) |  |
|  | Lee et al. (2012) [29] | III | 924 | Vandetanib 300 mg | 60 | 14.4 weeks | 1.9 | 619 | 164 (31) | 5 |
|  |  |  |  | Placebo | 60 | 10.7 weeks | 1.8 | 303 | 9 (0) |  |
|  | Ahn et al. (2013) [30] | II | 118 | Vandetanib 300 mg | 61 | 59 days | 2.7 | 75 | 13 (5) | 3 |
|  |  |  |  | Placebo | 60.5 | 54 days | 1.7 | 42 | 0 (0) |  |
|  | Heist et al. (2014) [31] | II | 130 | Sunitinib 37.5 mg + Pemetrexed | 63 | NR | 3.7 | 41 | (2) | 4 |
|  |  |  |  | Sunitinib 37.5 mg | 63 | NR | 3.3 | 47 | (2) |  |
|  |  |  |  | Pemetrexed | 63 | NR | 4.9 | 42 | (1) |  |
|  | Laurie et al. (2014) [32] | III | 306 | Cediranib 20 mg | 63 | 19.7 weeks | 5.5 | 151 | 51 (15) | 4 |
|  |  |  |  | Placebo | 62 | 19.1 weeks | 5.5 | 153 | 14 (3) |  |
|  | Gridelli et al. (2014) [33] | II | 124 | Vandetanib 100 mg + Gemcitabine | 75.03 | 78.5 days | 183 days | 61 | 0 (0) | 3 |
|  |  |  |  | Placebo + Gemcitabine | 75.48 | 91 days | 169 days | 63 | 0 (0) |  |
|  | Paz-Ares et al. (2015) [34] | III | 703 | Sorafenib 400 mg + BSC | 59 | 12 weeks | 2.8 | 346 | 68 (19) | 5 |
|  |  |  |  | Placebo + BSC | 62 | 6.3 weeks | 1.4 | 351 | 16 (2) |  |
| Breast cancer | Barrios et al. (2010) [35] | III | 482 | Sunitinib 37.5 mg | 53 | 61 days | 2.8 | 238 | 46 (9) | 3 |
|  |  |  |  | Capecitabine | 53 | 61 days | 4.2 | 240 | 1 (0) |  |
|  | Wildiers et al. (2010) [36] | II | 56 | Sunitinib 37.5 mg | 58 | NR | 2.8 | 36 | 17 (0) | 3 |
|  |  |  |  | No therapy | 59 | NR | 3.1 | 19 | 0 (0) |  |
|  | Rugo et al. (2011) [37] | II | 168 | Axitinib + TXT | 55 | NR | 8.1 | 111 | 31 (5) | 5 |
|  |  |  |  | Placebo + TXT | 56 | NR | 7.1 | 56 | 0 (0) |  |
|  | Bergh et al. (2012) [38] | III | 593 | Sunitinib 37.5 mg + TXT | 54 | 26 weeks | 8.6 | 295 | 36 (5) | 3 |
|  |  |  |  | TXT | 56 | 18 weeks | 8.3 | 293 | 3 (0) |  |
|  | Baselga et al. (2012) [39] | IIb | 229 | Sorafenib 400 mg + capecitabine | 55.1 | NR | 6.4 | 112 | 18 (1) | 5 |
|  |  |  |  | Placebo + capecitabine | 54.4 | NR | 4.1 | 112 | 12 (2) |  |
|  | Schwartzberg et al. (2013) [40] | IIb | 160 | Sorafenib 400 mg + GC | 53.5 | NR | 3.4 | 79 | 20 (4) | 5 |
|  |  |  |  | Placebo + GC | 54.2 | NR | 2.7 | 77 | 9 (1) |  |
|  | Crown et al. (2013) [41] | III | 442 | Sunitinib 37.5 mg + Capecitabine | 52 | 114/121 days | 5.5 | 217 | 47 (5) | 3 |
|  |  |  |  | Capecitabine | 54 | 143 days | 5.9 | 215 | 8 (2) |  |
|  | Johnston  et al. (2013) [42] | II | 150 | Pazopanib 400 mg + Lapatinib | 50 | NR | NR | 76 | 20 (4) | 4 |
|  |  |  |  | Lapatinib | 54 | NR | NR | 73 | 3 (0) |  |
|  | Curigliano et al. (2013) [43] | II | 217 | Sunitinib 37.5 mg | 52 | NR | 2 | 110 | 26 (3) | 3 |
|  |  |  |  | SOC | 52 | NR | 2.7 | 103 | 4 (0) |  |
| HCC | Llovet et al. (2008) [44] | III | 602 | Sorafenib 400 mg | 64.9 ± 11.2 | 5.3 | 5.5 | 297 | 5 (2) | 5 |
|  |  |  |  | Placebo | 66.3 ± 10.2 | 4.3 | 2.8 | 302 | 2 (1) |  |
|  | Cheng et al. (2009) [45] | III | 226 | Sorafenib 400 mg | 51 | NR | 2.8 | 149 | 28 (3) | 5 |
|  |  |  |  | Placebo | 52 | NR | 1.4 | 75 | 1 (0) |  |
|  | Abou-Alfa et al. (2010) [46] | II | 96 | Sorafenib 400 mg + doxorubicin | 66 | 119.7 days | 6 | 47 | 8 (0) | 5 |
|  |  |  |  | Placebo + doxorubicin | 65 | 56.7 days | 2.7 | 49 | 0 (0) |  |
|  | Kudo et al. (2011) [47] | III | 458 | Sorafenib 400 mg | 69 | 17.1 weeks | 5.4 | 229 | 31 (15) | 3 |
|  |  |  |  | Placebo | 70 | 20.1 weeks | 3.7 | 227 | 7 (1) |  |
|  | Hsu et al. (2012) [48] | II | 67 | Vandetanib 300 mg | 54 | 39 days | 1.05 | 19 | 2 | 5 |
|  |  |  |  | Vandetanib 100 mg | 61 | 43 days | 1.7 | 25 | 5 |  |
|  |  |  |  | Placebo | 56 | 30 days | 0.95 | 23 | 1 |  |
|  | Bruix et al. (2015) [49] | III | 1114 | Sorafenib 400 mg | 58 | 12.5 | NR | 559 | 108 (24) | 5 |
|  |  |  |  | Placebo | 60 | 22.2 | NR | 548 | 35 (6) |  |
|  | Lencioni et al. (2016) [50] | II | 307 | Sorafenib 400 mg + DEB-TACE | 64.5 | 21 weeks | 169 days | 153 | 46 (25) | 4 |
|  |  |  |  | Placebo + DEB-TACE | 63 | 27.3 weeks | 166 days | 151 | 25 (14) |  |
| RCC | Escudier et al. (2007) [51] | III | 903 | Sorafenib 400 mg | 58 | 23 weeks | 5.5 | 451 | 76 (16) | 5 |
|  |  |  |  | Placebo | 59 | 12 weeks | 2.8 | 451 | 8 (2) |  |
|  | Motzer et al. (2007) [52] | III | 750 | Sunitinib 50 mg | 62 | 6 | 11 | 375 | 24 (8) | 3 |
|  |  |  |  | Interferon alfa | 59 | 4 | 5 | 360 | 1 (1) |  |
|  | Sternberg et al. (2010) [53] | III | 435 | Pazopanib 800 mg | 59 | 7.4 | 9.2 | 290 | 115 (13) | 5 |
|  |  |  |  | Placebo | 60 | 3.8 | 4.2 | 145 | 15 (1) |  |
|  | Mulders et al. (2012) [54] | II | 71 | Cediranib 45 mg | 60 | 12 weeks | 12.1 | 53 | 34 (10) | 5 |
|  |  |  |  | Placebo | 61 | NR | 2.8 | 18 | 4 (0) |  |
|  | Haas et al. (2016) [55] | III | 1943 | Sunitinib 50 mg | 56 | 48 weeks | NR | 625 | (105) | 5 |
|  |  |  |  | Sorafenib 400 mg | 55 | 48 weeks | NR | 628 | (102) |  |
|  |  |  |  | Placebo | 57 | 54 weeks | NR | 626 | (26) |  |
| Thyroid cancer | Leboulleux et al. (2012) [56] | II | 145 | Vandetanib 300 mg | 63 | 192 days | 11.1 | 72 | 25 (0) | 5 |
|  |  |  |  | Placebo | 64 | 175.5 days | 5.9 | 73 | 4 (0) |  |
|  | Wells et al. (2012) [57] | III | 331 | Vandetanib 300 mg | 50.7 | 90.1 weeks | 30.5 | 231 | 73 (20) | 4 |
|  |  |  |  | Placebo | 53.4 | 39.9 weeks | 19.3 | 99 | 5 (0) |  |
|  | Elisei et al. (2013) [58] | III | 330 | Cabozantinib 140 mg | 55 | 204 days | 11.2 | 214 | 70 (18) | 3 |
|  |  |  |  | Placebo | 55 | 105 days | 4 | 109 | 5 (1) |  |
|  | Brose et al. (2014) [59] | III | 419 | Sorafenib 400 mg | 63 | 10.6 | 10.8 | 207 | 84 (20) | 5 |
|  |  |  |  | Placebo | 63 | 6.5 | 5.8 | 209 | 26 (5) |  |
| Pancreatic cancer | Spano et al. (2008) [60] | II | 103 | Axitinib 5 mg + gemcitabine | 65 | 113 days | 4.2 | 68 | 15 (4) | 3 |
|  |  |  |  | Gemcitabine | 61 | 4 cycles | 3.7 | 31 | 0 (0) |  |
|  | Kindler et al. (2011) [61] | III | 632 | Axitinib 5 mg + gemcitabine | 61 | 2.8 | 4.4 | 305 | 85 (20) | 5 |
|  |  |  |  | Placebo + gemcitabine | 62 | 2.3 | 4.4 | 308 | 27 (5) |  |
|  | GonÇalves et al. (2012) [62] | III | 104 | Sorafenib 200 mg + Gemcitabine | 61 | NR | 3.8 | 50 | 4 (0) | 4 |
|  |  |  |  | Placebo + Gemcitabine | 64 | NR | 5.7 | 52 | 4 (2) |  |
|  | Reni et al. (2013) [63] | II | 56 | Sunitinib 37.5 mg | 61 | 91 days | 3.2 | 27 | 3 (0) | 3 |
|  |  |  |  | Observation | 65 | NR | 2.0 | 28 | 0 (0) |  |
| mCRC | Hoff et al. (2012) [64] | III | 1076 | Cediranib 20 mg + FOLFOX/CAPOX | 58 | NR | 8.6 | 500 | 230 (55) | 3 |
|  |  |  |  | Placebo + FOLFOX/CAPOX | 59 | NR | 8.3 | 358 | 43 (7) |  |
|  | Grothey et al. (2013) [65] | III | 760 | Regorafenib 160 mg | 61 | 2.8 | 1.9 | 500 | 139 (36) | 5 |
|  |  |  |  | Placebo | 61 | 1.8 | 1.7 | 253 | 15 (2) |  |
|  | Tabernero et al. (2013) [66] | IIb | 198 | Sorafenib 400 mg + FOLFOX6 | 59.2 | 30.5 weeks | 9.1 | 97 | 18 (4) | 4 |
|  |  |  |  | Placebo + FOLFOX 6 | 101 | 33.7 weeks | 8.7 | 101 | 5 (1) |  |
|  | Carrato et al. (2013) [67] | III | 768 | Sunitinib 37.5 mg + FOLFIRI | 59 | NR | 7.8 | 384 | (10) | 3 |
|  |  |  |  | Placebo + FOLFIRI | 58 | NR | 8.4 | 379 | (3) |  |
| Ovarian cancer | Herzog et al. (2013) [68] | IIB | 246 | Sorafenib 400 mg | 56.9 | 17.6 weeks | 12.7 | 123 | 45 (10) | 3 |
|  |  |  |  | Placebo | 54.4 | 51.9 weeks | 15.7 | 123 | 7 (1) |  |
|  | du Bois et al. (2014) [69] | III | 940 | Pazopanib 800 mg | 56 | 8.9 | 17.9 | 477 | 275 (147) | 5 |
|  |  |  |  | Placebo | 57 | 11.7 | 12.3 | 461 | 91 (26) |  |
|  | Pignata et al. (2015) [70] | II | 74 | Pazopanib 800 mg + paclitaxel | 56 | NR | 6.35 | 37 | 16 (3) | 4 |
|  |  |  |  | Paclitaxel | 58 | NR | 3.49 | 36 | 0 (0) |  |
|  | Hainsworth et al. (2015) [71] | II | 85 | Sorafenib 400 mg + CP | 63 | NR | 15.4 | 43 | 10 (2) | 3 |
|  |  |  |  | CP | 62 | NR | 16.3 | 42 | 2 (0) |  |
| GIST | Demetri et al. (2012) [72] | III | 361 | Sunitinib 50 mg | 57 | 12 weeks | 22.9 weeks | 228 | 27 (9) | 5 |
|  |  |  |  | Placebo | 55 | 6 weeks | 6.0 weeks | 114 | 5 (1) |  |
|  | Demetri et al. (2013) [73] | III | 240 | Regorafenib 160 mg | 60 | 22.9 weeks | 4.8 | 133 | 64 (31) | 5 |
|  |  |  |  | Placebo | 61 | 7.0 weeks | 0.9 | 66 | 11 (2) |  |
|  | Komatsu et al. (2015) [74] | III | 17 | Regorafenib 160 mg | 57 | 23.1 weeks | 7.1 | 12 | 6 (3) | 3 |
|  |  |  |  | Placebo | 49 | 7.9 weeks | 0.9 | 5 | 0 (0) |  |
| R/M HNSCC | Limaye et al. (2013) [75] | II | 30 | Vandetanib 100 mg + TXT | 60 | 63 days | 9 weeks | 15 | 0 (0) | 3 |
|  |  |  |  | TXT | 56 | 42 days | 3.21 weeks | 14 | 0 (0) |  |
|  | Gilbert et al. (2015) [76] | II | 55 | Sorafenib 400 mg + cetuximab | 28 | 65.1 days | 3.2 | 28 | (1) | 3 |
|  |  |  |  | Cetuximab | 27 | 86.1 days | 3 | 27 | (0) |  |
| Melanoma | McDermott et al. (2008) [77] | II | 121 | Sorafenib 400 mg + dacarbazine | 55 | 19.1 weeks | 21.1 weeks | 51 | 7 (4) | 5 |
|  |  |  |  | Placebo + dacarbazine | 60 | 12.1 weeks | 11.7 weeks | 50 | 3 (0) |  |
|  | Flaherty et al. (2013) [77] | III | 823 | Sorafenib 400 mg + CP | 61 | NR | 4.9 | 393 | (18) | 3 |
|  |  |  |  | Placebo + CP | 59 | NR | 4.2 | 397 | (5) |  |
| Prostate cancer | Horti et al. (2009) [78] | II | 86 | Vandetanib 100 mg + DP | 67 | NR | NR | 43 | 6 (0) | 5 |
|  |  |  |  | Placebo + DP | 67 | NR | NR | 43 | 1 (0) |  |
|  | Michaelson et al. (2014) [79] | III | 873 | Sunitinib 37.5 mg + Prednisone | 69 | 98 days | 5.6 | 581 | (24) | 5 |
|  |  |  |  | Placebo + Prednisone | 68 | 97 days | 4.1 | 285 | (1) |  |
| STS | Winette et al. (2012) [80] | III | 369 | Pazopanib 800 mg | 56.7 | 16.4 weeks | 4.6 | 239 | 99 (16) | 5 |
|  |  |  |  | Placebo | 51.9 | 8.1 weeks | 1.6 | 123 | 8 (4) |  |
|  | Kawai et al. (2016) [81] | III | 47 | Pazopanib 800 mg | 53.5 | 21.9 weeks | 24.7 weeks | 31 | 16 (5) | 5 |
|  |  |  |  | Placebo | 50.1 | 8.5 weeks | 7 weeks | 16 | 3 (0) |  |
| SCLC | Arnold et al. (2007) [82] | II | 107 | Vandetanib 300 mg | 56.9 | 7 weeks | 2.7 | 52 | 21 (2) | 5 |
|  |  |  |  | Placebo | 62.4 | 12 weeks | 2.8 | 53 | 9 (2) |  |
| PNET | Raymond et al. (2011) [83] | III | 171 | Sunitinib 37.5 mg | 56 | 4.6 | 11.4 | 83 | 22 (8) | 5 |
|  |  |  |  | Placebo | 57 | 3.7 | 5.5 | 82 | 4 (1) |  |
| Urothelial cancer | Krege et al. (2014) [84] | II | 132 | Sorafenib 400 mg + GC | 64.4 | NR | 6.3 | 40 | 3 (1) | 4 |
|  |  |  |  | GC | 67.3 | NR | 6.1 | 49 | 1 (0) |  |
| Gastric cancer | Yi et al. (2012) [85] | II | 105 | Sunitinib 37.5 mg + TXT | 54 | NR | 3.9 | 56 | 0 (0) | 3 |
|  |  |  |  | TXT | 52 | NR | 2.6 | 49 | 0 (0) |  |
| AML | Rollig et al. (2015) [86] | II | 276 | Sorafenib 400 mg + cytarabine | 50 | NR | 21 | 134 | 29 (3) | 5 |
|  |  |  |  | Cytarabine | 50 | NR | 9 | 133 | 13 (1) |  |
| Biliary tract cancer | Santoro et al. (2014) [87] | II | 174 | Vandetanib 100 mg + gemcitabine | 64.4 | 78 days | 114 days | 58 | 8 (2) | 3 |
|  |  |  |  | Vandetanib 300 mg | 62.4 | 45 days | 105 days | 59 | 13 (4) |  |
|  |  |  |  | Placebo + gemcitabine | 64 | 75.5 days | 148 days | 56 | 4 (1) |  |

TXT, docetaxel; NR, not reported; CP, paclitaxel plus carboplatin; GC, gemcitabine plus cisplatin; BSC, best supportive care; SOC, standard-of-care; DEB-TACE, transarterial chemoembolization with doxorubicin-eluting beads; FOLFOX, fluorouracil in combination with leucovorin and oxaliplatin; FOLFIRI, fluorouracil in combination with leucovorin and irinotecan; CAPOX, capecitabine plus oxaliplatin; DP, docetaxel plus prednisolone; NSCLC, non-small cell lung cancer; HCC, hepatocellular carcinoma; RCC, renal-cell carcinoma; mCRC, metastatic colorectal cancer; GIST, gastrointestinal stromal tumor; R/M HNSCC, recurrent and/or metastatic head and neck squamous cell carcinoma; STS, soft-tissue sarcoma; SCLC, small-cell lung cancer; PNET; pancreatic neuroendocrine tumors; AML, acute myeloid leukemia.
