# Supplementary material for: Incidence and risk of hypertension associated with vascular endothelial growth factor receptor tyrosine kinase inhibitors in cancer patients: a comprehensive network meta-analysis of 72 randomized controlled trials involving 30013 patients
Source: Oncotarget. 2016 Sep 1;7(41):67661–73. doi: 10.18632/oncotarget.11813 (PMC5341903; doi:10.18632/oncotarget.11813)
Supplement: Supplementary file 5 [file oncotarget-07-67661-s005.doc]

**Supplemental Table 4.** Incidence and relative risk of hypertensive events associated with VEGFR-TKIs according to tumor type, VEGFR-TKI, trial phase, chemotherapy conditions, VEGFR-TKI-based regimen, and control therapy

| Groups | Studies, *n* | All-grade hypertension, *n*/Incidence, % | | RR (95% CI)^1^ | *P* value | Studies, *n* | High-grade hypertension, *n*/Incidence, % | | RR (95% CI)^2^ | *P* value |
| --- | --- | --- | --- | --- | --- | --- | --- | --- | --- | --- |
|  |  | VEGFR-TKI | Control |  |  |  | VEGFR-TKI | Control |  |  |
| Overall | 64 | 2972/23.0 | 610/3.7 | 3.85 (3.37-4.40) | <0.001 | 71 | 1023/4.4 | 156/0.3 | 4.60 (3.92-5.40) | <0.001 |
| Tumor  type |  |  |  |  |  |  |  |  |  |  |
| NSCLC | 15 | 816/17.4 | 175/4.2 | 3.95 (3.12-5.00) | <0.001 | 18 | 184/3.5 | 28/0.6 | 5.23 (3.56-7.67) | <0.001 |
| Breast cancer | 9 | 261/22.5 | 40/3.4 | 6.15 (2.96-12.79) | <0.001 | 9 | 36/2.8 | 5/0.4 | 4.40 (2.07-9.38) | <0.001 |
| HCC | 7 | 233/16.4 | 71/5.2 | 3.04 (2.36-3.92) | <0.001 | 6 | 69/4.8 | 22/1.6 | 2.94 (1.86-4.64) | <0.001 |
| RCC | 4 | 249/30.6 | 28/2.9 | 5.55 (2.75-11.19) | <0.001 | 5 | 254/10.5 | 30/1.9 | 4.42 (3.07-6.37) | <0.001 |
| Thyroid cancer | 4 | 252/34.6 | 40/8.2 | 4.61 (3.34-6.38) | <0.001 | 4 | 58/8 | 6/1.2 | 6.02 (2.19-11.07) | <0.001 |
| Pancreatic cancer | 4 | 107/17.6 | 31/7.4 | 3.22 (2.21-4.69) | 0.008 | 4 | 24/5.3 | 7/1.7 | 2.79 (1.28-6.07) | 0.010 |
| mCRC | 3 | 387/31.0 | 63/8.8 | 4.05 (3.16-5.20) | <0.001 | 4 | 105/7.1 | 13/1.2 | 5.68 (3.17-10.16) | <0.001 |
| Ovarian cancer | 4 | 346/40.6 | 100/15.1 | 4.65 (2.30-9.42) | <0.001 | 4 | 162/23.8 | 27/4.1 | 5.64 (3.84-8.27) | <0.001 |
| GIST | 3 | 97/35.4 | 16/8.6 | 2.93 (1.82-4.72) | <0.001 | 3 | 43/11.5 | 3/1.6 | 6.13 (2.11-17.80) | 0.001 |
| R/M HNSCC | 1 | 0/0 | 0/0 | 0.94 (0.02-44.33) | 0.974 | 2 | 1/2.3 | 0/0 | 1.91 (0.18-20.27) | 0.591 |
| Melanoma | 1 | 7/13.7 | 3/6 | 2.29 (0.63-8.35) | 0.210 | 2 | 22/5 | 5/1.1 | 4.12 (1.63-10.37) | 0.003 |
| Prostate cancer | 1 | 6/14 | 1/2.3 | 6.00 (0.75-47.76) | 0.090 | 2 | 24/3.8 | 1/0.3 | 8.85 (1.59-49.12) | 0.013 |
| STS | 2 | 115/42.6 | 11/7.9 | 5.38 (3.01-9.64) | <0.001 | 2 | 21/7.8 | 4/2.9 | 2.48 (0.92-6.69) | 0.074 |
| SCLC | 1 | 21/40.4 | 9/17 | 2.38 (1.20-4.70) | 0.013 | 1 | 2/3.8 | 2/3.8 | 1.02 (0.15-6.97) | 0.985 |
| PNET | 1 | 22/26.5 | 4/4.9 | 5.43 (1.96-15.08) | 0.001 | 1 | 8/9.6 | 1/1.2 | 7.90 (1.01-61.79) | 0.049 |
| Urothelial cancer | 1 | 3/7.5 | 1/2 | 3.67 (0.40-33.98) | 0.251 | 1 | 1/2.5 | 0/0 | 3.66 (0.15-87.44) | 0.423 |
| Gastric cancer | 1 | 0/0 | 0/0 | 0.88 (0.02-43.40) | 0.948 | 1 | 0/0 | 0/0 | 0.88 (0.02-43.40) | 0.948 |
| AML | 1 | 29/21.6 | 13/9.8 | 2.21 (1.21-4.70) | 0.010 | 1 | 3/2.2 | 1/0.8 | 2.98 (0.31-28.26) | 0.342 |
| Biliary tract cancer | 1 | 21/17.9 | 4/7.1 | 2.51 (0.91-6.97) | 0.077 | 1 | 6/5.1 | 1/1.8 | 2.87 (0.35-23.29) | 0.323 |
| VEGFR-TKI |  |  |  |  |  |  |  |  |  |  |
| Sorafenib | 21 | 741/18.1 | 228/5.7 | 3.07 (2.43-3.87) | <0.001 | 24 | 307/6 | 80/1.6 | 3.66 (2.89-4.63) | <0.001 |
| Vandetanib | 15 | 540/17.3 | 72/3 | 5.25 (4.12-6.70) | <0.001 | 15 | 118/3.7 | 11/0.4 | 5.85 (3.36-10.20) | <0.001 |
| Sunitinib | 10 | 248/14.9 | 26/1.7 | 7.91 (5.40-11.57) | <0.001 | 15 | 194/5.1 | 38/1.1 | 4.35 (3.12-6.07) | <0.001 |
| Pazopanib | 6 | 541/47 | 38/4.4 | 7.58 (3.08-18.62) | <0.001 | 6 | 188/16.3 | 31/3.6 | 5.06 (3.55-7.22) | <0.001 |
| Cediranib | 4 | 353/42.5 | 71/10.9 | 3.72 (2.95-4.70) | <0.001 | 4 | 99/11.9 | 12/1.8 | 6.13 (3.43-10.97) | <0.001 |
| Axitinib | 3 | 131/27.1 | 27/6.8 | 9.17 (0.72-116.54) | 0.087 | 3 | 29/6 | 5/1.3 | 4.22 (1.75-10.16) | 0.001 |
| Regorafenib | 3 | 209/32.4 | 26/8 | 3.96 (2.72-5.79) | <0.001 | 3 | 70/10.9 | 4/1.2 | 7.81 (3.06-19.94) | <0.001 |
| Motesanib | 1 | 139/26.1 | 35/6.5 | 4.02 (2.83-5.70) | <0.001 | 1 | 0/0 | 0/0 | 1.01 (0.02-50.87) | 0.996 |
| Cabozantinib | 1 | 70/32.7 | 5/4.6 | 7.13 (2.97-17.15) | <0.001 | 1 | 18/8.4 | 1/0.9 | 9.17 (1.24-67.77) | 0.030 |
| Phases of trials |  |  |  |  |  |  |  |  |  |  |
| Phase II | 31 | 554/20.2 | 121/1.6 | 3.43 (2.66-4.42) | <0.001 | 33 | 143/2.2 | 28/0.1 | 3.28 (2.31-4.66) | <0.001 |
| Phase III | 33 | 2418/24.6 | 489/4.9 | 4.06 (3.48-4.74) | <0.001 | 38 | 880/5.5 | 128/0.6 | 4.97 (4.14-5.96) | <0.001 |
| Chemotherapy condition |  |  |  |  |  |  |  |  |  |  |
| Chemotherapy-naïve | 31 | 1304/21.6 | 310/4.3 | 3.33 (2.82-3.94) | <0.001 | 33 | 523/4.2 | 90/0.4 | 3.87 (3.12-4.81) | <0.001 |
| Pre-Chemotherapy | 33 | 1668/24.8 | 300/3.4 | 4.36 (3.57-5.33) | <0.001 | 38 | 500/4.8 | 66/0.3 | 5.61 (4.40-7.16) | <0.001 |
| VEGFR-TKI-based regimens |  |  |  |  |  |  |  |  |  |  |
| VEGFR-TKIs alone | 33 | 1840/28.4 | 317/4.1 | 4.49 (3.74-5.39) | <0.001 | 35 | 728/6.6 | 91/0.4 | 5.29 (4.30-6.51) | <0.001 |
| Combinations | 33 | 1132/17.8 | 299/3.5 | 3.24 (2.70-3.88) | <0.001 | 39 | 312/2.8 | 67/0.3 | 3.80 (2.96-4.88) | <0.001 |
| Control therapy |  |  |  |  |  |  |  |  |  |  |
| Placebo | 25 | 1598/31.3 | 289/5.8 | 4.16 (3.47-4.98) | <0.001 | 25 | 666/8.4 | 85/0.7 | 5.13 (4.13-6.38) | <0.001 |
| Non-placebo | 39 | 1374/17.9 | 321/2.8 | 3.63 (2.98-4.41) | <0.001 | 46 | 357/2.5 | 74/0.2 | 3.81 (3.01-4.83) | <0.001 |

^1^*P* < 0.001 for variation in relative risk (RR) according to tumor type; *P* < 0.001 for variation in RR according to VEGFR-TKI; *P* < 0.001 for variation in RR according to trial phase; *P* < 0.001 for variation in RR according to chemotherapy conditions; *P* < 0.001 for variation in RR according to VEGFR-TKI-based regimen; *P* < 0.001 for variation in RR according to controlled therapy.

^2^*P* < 0.001 for variation in RR according to tumor type; *P* < 0.001 for variation in RR according to VEGFR-TKI; *P* < 0.001 for variation in RR according to trial phase; *P* < 0.001 for variation in RR according to chemotherapy conditions; *P* < 0.001 for variation in RR according to VEGFR-TKI-based regimen; *P* < 0.001 for variation in RR according to control therapy.

NSCLC, non-small cell lung cancer; HCC, hepatocellular carcinoma; RCC, renal-cell carcinoma; mCRC, metastatic colorectal cancer; GIST, gastrointestinal stromal tumor; R/M HNSCC, recurrent and/or metastatic head and neck squamous cell carcinoma; STS, soft-tissue sarcoma; SCLC, small-cell lung cancer; PNET; pancreatic neuroendocrine tumors; AML, acute myeloid leukemia.
